# The Surgical Treatment of Hypertension—II
*See footnote, page 103.


**Published:** 1950-10

**Authors:** Ashton Miller


### THE SURGICAL TREATMENT OF HYPERTENSION—II *

BY

ASHTON MILLER, F.R.C.S.

Early in 1947 the Surgical Professorial Unit of the Bristol Royal Hospital under the direction of Professor Milnes Walker started the surgical treatment of cases of hypertension. This report concerns thirteen patients who were operated upon in 1947 and 1948, and whose post-operative course has been followed to the present day. Cases dealt with in 1949 and 1950 have been excluded from this discussion. The operations have been performed by all three members of the Unit, but substantially the same technique has been used in each case. It was decided at the beginning that when selecting cases for surgical treatment the same indications should be used, which were as follows:

1. Malignant Hypertension—under fifty years of age, and without cardiac or renal failure.

2. Essential Hypertension—under fifty years of age, a history of less than five years, marked symptoms, and no cardiac or renal failure.

This has excluded:

A. All cases of benign essential hypertension which simply have a high blood pressure and no symptoms associated with it,

B. All cases with a diastolic blood pressure persistently less than 120 mm. of mercury, and

C. All cases of renal hypertension due to a unilateral renal lesion.

### TECHNIQUE

The Smithwick type of operation has been performed in all cases, first on one side and then on the other, with an interval

* See footnote, page 103.

| Case Number | Sex | Age | Duration of Symptoms (months) | Headache | Vertigo | Nausea and Vomiting | Other Symptoms | B.P. (average) | Cardiac Enlargement | E.C.G. Changes | Renal Failure | Fundi, Grade |
|---|---|---|---|---|---|---|---|---|---|---|---|---|
| *Malignant Hypertension* | | | | | | | | | | | | |
| I | F | 18 | — | — | — | — | — | 230/150 | — | — | — | IV |
| II | F | 19 | — | — | — | — | — | 210/170 | — | — | — | IV |
| III | M | 52 | 2 | + | + | — | — | 230/150 | + | + | — | IV |
| IV | M | 45 | 4 | + | + | + | + | 250/150 | — | — | — | IV |
| V | M | 40 | 6 | + | + | + | — | 220/140 | + | — | — | IV |
| VI | F | 43 | 7 | + | + | + | + | 225/160 | — | — | — | IV |
| VII | F | 46 | 3 | + | + | — | + | 240/120 | — | — | — | IV |
| *Benign Essential Hypertension* | | | | | | | | | | | | |
| VIII | M | 43 | 60 | + | + | — | — | 210/150 | — | — | — | II |
| IX | F | 46 | 72 | + | + | + | — | 260/140 | — | — | — | II |
| X | F | 43 | 30 | + | + | — | + | 240/120 | — | — | — | II |
| XI | F | 46 | 72 | + | — | + | + | 215/130 | — | — | — | II |
| *Post-Eclamptic Hypertension* | | | | | | | | | | | | |
| XII | F | 27 | 5 | + | + | + | + | 230/150 | — | — | — | IV |
| XIII | Untraced | | | | | | | | | | | |

| Time Since Operation (months) | Symptoms | Cardiac Enlargement | E.C.G. Changes | B.P. (in O.P.D.) | Fundi | |
|---|---|---|---|---|---|---|
| 18 | — | — | — | 260/150 | I | At work. |
| 24 | — | + | + | 170/140 | I | Died of cerebral haemorrhage twenty-six months after operation. |
| | — | + | + | 240/160 | II | Post-operative Empyema. Died of renal failure ten months after operation. |
| 16 | — | — | — | 230/130 | I | At work. |
| 18 | — | + | — | 200/112 | I | At work. |
| 15 | — | — | — | 180/120 | I | Working as housewife. |
| 26 | — | + | — | 160/100 | I | Working as housewife. |
| 17 | ± | — | — | 190/130 | II | Headaches tolerable. At work. |
| 18 | + | + | — | 260/160 | II | Headaches tolerable, but cannot work. |
| 20 | — | — | + | 160/106 | I | Working as housewife. |
| | + | — | — | 200/110 | II | Died seven months later of cerebral thrombosis and bronchopneumonia. |
| 18 | — | — | — | 200/120 | I | Working as housewife. |

of about two weeks. At first the approach was extrapleural, but recently the quicker transpleural route has been used in all cases. The sympathetic chain is removed from the ninth dorsal to the second lumbar ganglion inclusive and the splanchnic nerves found and removed. There has been no fatality attributable to the operation, but certain post-operative complications have occurred which are the result of the thoracotomy. Pleural effusion needing aspiration was met with in two of the thirteen patients while one of these went on to form an empyema which needed open drainage. Lobar collapse has occurred but has responded to active physio-therapy. Persistent post-operative intercostal pain has been seen in several cases, but has never been severe and has never lasted more than two months.

### Results

*Malignant Hypertension.* The two younger, almost symptomless cases show no significant change, apart from the fundi which have almost returned to normal in both patients. (Since the date of the meeting, one of these patients has died of cerebral haemorrhage.) Of the older group of five patients (three men, two women), there are four survivors whose symptoms have been improved so that they are able to do some kind of work. Their fundi all show great improvement, but their blood pressures are all substantially the same. One patient shows electrocardiographic signs of early cardiac strain, and another shows cardiac enlargement.

*Benign Essential Hypertension.* Of the four patients included in this group one is undoubtedly improved and is able to do work which was impossible before operation: her blood pressure is lower but has not fallen to within normal limits. Another is unchanged after operation and a third improved as regards symptoms and blood pressure, but showing signs of cardiac strain on the electrocardiogram. It is worth noting that all these patients were convinced that the operation had been worth while.

*Post-eclamptic Hypertension.* This patient shows a significant improvement in symptoms and fundi, combined with a lowering of blood pressure which has persisted for a year and a half.

### IMPRESSIONS

(*a*) Dorso-lumbar sympathectomy for hypertension consists of two major operations carrying a significant risk if cardiac or renal failure is present, but with little risk in earlier cases. Such a procedure has, of course, enormous value as a means of suggestion but we are of the opinion that the results are not entirely due to this. If they were it would be unnecessary to operate upon more than one side.

(*b*) Almost all the patients that are referred to hospital for consideration of surgical treatment for hypertension can be placed in two large groups: on the one hand those too bad for surgery because of cardiac or renal failure and on the other hand those whose symptoms are not bad enough. In between them come a small number consisting of:

1. Early cases of malignant hypertension.
2. Occasional cases of benign hypertension with a short history and severe incapacity.
3. A few cases where the marked and apparently certain improvement in the fundi can be expected to save rapidly failing sight.
4. Cases of post-eclamptic hypertension.

(*c*) The diastolic blood pressure has not been lowered below 100 mm. mercury in any case, and a significant persistent lowering of systolic and diastolic pressure has only been obtained in two cases out of the thirteen.

(*d*) In the great majority of cases symptoms can be relieved and the fundi brought back almost to normal. The reasons for this are unknown.

(*e*) We have no evidence as yet that this operation prolongs life.

